# *N*-Phenyl­cyclo­hexa­necarboxamide

**DOI:** 10.1107/S1600536810039267

**Published:** 2010-10-09

**Authors:** Bin Dong, Yu Zhang, Dong-Yu Wang

**Affiliations:** a Affiliated Hospital of Hebei University, Baoding 071000, People’s Republic of China; b Hebei Xushui County Health Bureau, Baoding 071000, People’s Republic of China; c College of Electronic and Information Engineering, Hebei University, Baoding 071000, People’s Republic of China

## Abstract

In the title compound, C_13_H_17_NO, the cyclo­hexane ring adopts a chair conformation and the amide C(=O)—N moiety is almost coplanar with the phenyl ring [C—N—C—O = 4.1 (2)°]. In the crystal, mol­ecules are linked to form a *C*(4) infinite [001] chain *via* N—H⋯O hydrogen bonds, unlike the cyclic motif seen in related structures.

## Related literature

For hydrogen-bonding motifs in amides, see: Taylor *et al.* (1984 ▶); Leiserowitz & Schmidt (1969 ▶). For related structures, see: Lemmerer & Michael (2008 ▶).
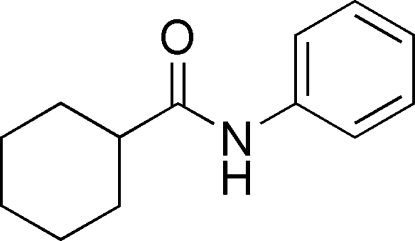


## Experimental

### 

#### Crystal data

C_13_H_17_NO
*M* *_r_* = 203.28
Orthorhombic, 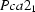

*a* = 9.943 (2) Å
*b* = 11.839 (2) Å
*c* = 9.6514 (19) Å
*V* = 1136.1 (4) Å^3^
*Z* = 4
Mo *K*α radiation
μ = 0.08 mm^−1^
*T* = 113 K
0.24 × 0.18 × 0.10 mm

#### Data collection

Rigaku Saturn CCD diffractometer
Absorption correction: multi-scan (*CrystalClear*; Rigaku/MSC, 2005 ▶) *T*_min_ = 0.982, *T*_max_ = 0.993
8926 measured reflections
1431 independent reflections
1308 reflections with *I* > 2σ(*I*)
*R*_int_ = 0.038

#### Refinement

*R*[*F* ^2^ > 2σ(*F* ^2^)] = 0.033
*wR*(*F* ^2^) = 0.080
*S* = 1.09
1431 reflections
141 parameters
1 restraint
H atoms treated by a mixture of independent and constrained refinement
Δρ_max_ = 0.14 e Å^−3^
Δρ_min_ = −0.12 e Å^−3^

### 

Data collection: *CrystalClear* (Rigaku/MSC, 2005 ▶); cell refinement: *CrystalClear*; data reduction: *CrystalClear*; program(s) used to solve structure: *SHELXS97* (Sheldrick, 2008 ▶); program(s) used to refine structure: *SHELXL97* (Sheldrick, 2008 ▶); molecular graphics: *SHELXTL* (Sheldrick, 2008 ▶); software used to prepare material for publication: *SHELXL97*.

## Supplementary Material

Crystal structure: contains datablocks I, global. DOI: 10.1107/S1600536810039267/hb5665sup1.cif

Structure factors: contains datablocks I. DOI: 10.1107/S1600536810039267/hb5665Isup2.hkl

Additional supplementary materials:  crystallographic information; 3D view; checkCIF report

## Figures and Tables

**Table 1:** Hydrogen-bond geometry (Å, °)

*D*—H⋯*A*	*D*—H	H⋯*A*	*D*⋯*A*	*D*—H⋯*A*
N1—H1⋯O1^i^	0.85 (3)	1.98 (3)	2.8145 (19)	171.7 (18)

Symmetry code: (i) 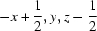.

**Fig. 1.**
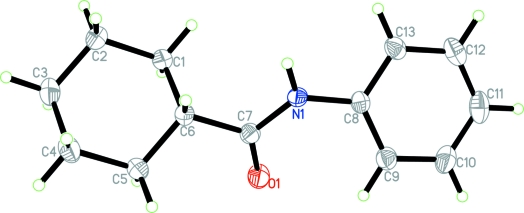
The molecule of (I) showing displacement ellipsoids drawn at the 50% probability level.

**Fig. 2.**
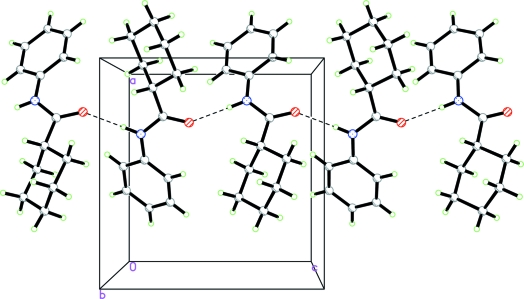
The infinite chain formed via N—H···O down the c axis.

## supplementary crystallographic information

### Comment

The amides are an important H-bonding supramolecular synthon (Taylor *et al.*, 1984; Leiserowitz & Schmidt, 1969), and we herein report the crystal structure of the title compound (I).

In the crystal structure of the title compound, Fig. 1, the cyclohexane group adopts a chair conformation [torsion angles: C1/C2/C3/C4 54.67 (19)°, C2/C3/C4/C5 - 55.3 (2)°]. The amide C(=O)—N moiety is almost coplanar with the phenyl ring [torsion angles: C8/N1/C7/O1 4.1 (2)°, C8/N1/C7/C6 - 175.38 (13)°]. Molecules are linked to form an infinite chain down the *c* axis *via* N—H···O hydrogen bonds (Fig. 2 and Table 1), being different from the reported secondary graph set *R*_6_^4^(16) in 1-phenylcylcopentane- carboxamide and 1-(2-bromphenyl)cyclopentanecarboxamide (Lemmerer & Michael, 2008).

### Experimental

The title compound was prepared from cyclohexoyl chloride and aniline. Colourless blocks of (I) were grown out *via* recrystallization from ethanol.

### Refinement

Anomalous dispersion was negligible and Friedel pairs were merged before refinement. The amide H atom was located in a difference Fourier map and refined freely. The other H atoms were positioned geometrically and allowed to ride on their parent atoms [C—H = 1.00 (aliphic CH), 0.95(aromatic CH) or 0.99Å (CH_2_), and *U*_iso_(H) = 1.2 *U*_eq_(C)]

### Crystal data

**Table:**

C_13_H_17_NO	*F*(000) = 440
*M**_r_* = 203.28	*D*_x_ = 1.188 Mg m^−^^3^
Orthorhombic, *P**c**a*2_1_	Mo *K*α radiation, λ = 0.71073 Å
Hall symbol: P 2c -2ac	Cell parameters from 3664 reflections
*a* = 9.943 (2) Å	θ = 2.9–27.8°
*b* = 11.839 (2) Å	µ = 0.08 mm^−^^1^
*c* = 9.6514 (19) Å	*T* = 113 K
*V* = 1136.1 (4) Å^3^	Block, colorless
*Z* = 4	0.24 × 0.18 × 0.10 mm

### Data collection

**Table:**

Rigaku Saturn CCD diffractometer	1431 independent reflections
Radiation source: rotating anode	1308 reflections with *I* > 2σ(*I*)
multilayer	*R*_int_ = 0.038
Detector resolution: 7.31 pixels mm^-1^	θ_max_ = 27.9°, θ_min_ = 3.4°
ω and φ scans	*h* = −13→11
Absorption correction: multi-scan (*CrystalClear*; Rigaku/MSC, 2005)	*k* = −15→13
*T*_min_ = 0.982, *T*_max_ = 0.993	*l* = −12→12
8926 measured reflections	

### Refinement

**Table:**

Refinement on *F*^2^	Secondary atom site location: difference Fourier map
Least-squares matrix: full	Hydrogen site location: inferred from neighbouring sites
*R*[*F*^2^ > 2σ(*F*^2^)] = 0.033	H atoms treated by a mixture of independent and constrained refinement
*wR*(*F*^2^) = 0.080	*w* = 1/[σ^2^(*F*_o_^2^) + (0.0508*P*)^2^ + 0.0154*P*] where *P* = (*F*_o_^2^ + 2*F*_c_^2^)/3
*S* = 1.09	(Δ/σ)_max_ = 0.001
1431 reflections	Δρ_max_ = 0.14 e Å^−^^3^
141 parameters	Δρ_min_ = −0.12 e Å^−^^3^
1 restraint	Extinction correction: *SHELXL97* (Sheldrick, 2008), Fc^*^=kFc[1+0.001xFc^2^λ^3^/sin(2θ)]^-1/4^
Primary atom site location: structure-invariant direct methods	Extinction coefficient: 0.174 (16)

### Special details

**Table:**

Geometry. All e.s.d.'s (except the e.s.d. in the dihedral angle between two l.s. planes) are estimated using the full covariance matrix. The cell e.s.d.'s are taken into account individually in the estimation of e.s.d.'s in distances, angles and torsion angles; correlations between e.s.d.'s in cell parameters are only used when they are defined by crystal symmetry. An approximate (isotropic) treatment of cell e.s.d.'s is used for estimating e.s.d.'s involving l.s. planes.
Refinement. Refinement of *F*^2^ against ALL reflections. The weighted *R*-factor *wR* and goodness of fit *S* are based on *F*^2^, conventional *R*-factors *R* are based on *F*, with *F* set to zero for negative *F*^2^. The threshold expression of *F*^2^ > σ(*F*^2^) is used only for calculating *R*-factors(gt) *etc*. and is not relevant to the choice of reflections for refinement. *R*-factors based on *F*^2^ are statistically about twice as large as those based on *F*, and *R*- factors based on ALL data will be even larger.

### Fractional atomic coordinates and isotropic or equivalent isotropic displacement parameters (Å^2^)

**Table:**

	*x*	*y*	*z*	*U*_iso_*/*U*_eq_	
O1	0.22562 (12)	0.24856 (10)	0.38081 (13)	0.0311 (3)	
N1	0.17317 (13)	0.25866 (10)	0.15287 (15)	0.0201 (3)	
C1	0.48835 (17)	0.24779 (12)	0.16381 (19)	0.0254 (4)	
H1A	0.4512	0.2918	0.0854	0.031*	
H1B	0.5133	0.3014	0.2382	0.031*	
C2	0.61342 (18)	0.18387 (14)	0.11590 (18)	0.0282 (4)	
H2A	0.5904	0.1367	0.0346	0.034*	
H2B	0.6832	0.2387	0.0871	0.034*	
C3	0.66924 (17)	0.10871 (15)	0.2307 (2)	0.0344 (4)	
H3A	0.7032	0.1566	0.3071	0.041*	
H3B	0.7457	0.0642	0.1941	0.041*	
C4	0.56207 (18)	0.02845 (14)	0.2867 (2)	0.0334 (4)	
H4A	0.5996	−0.0153	0.3651	0.040*	
H4B	0.5356	−0.0255	0.2133	0.040*	
C5	0.43777 (16)	0.09411 (13)	0.33568 (18)	0.0259 (4)	
H5A	0.3681	0.0404	0.3679	0.031*	
H5B	0.4625	0.1434	0.4146	0.031*	
C6	0.38120 (15)	0.16622 (12)	0.21778 (17)	0.0218 (3)	
H6	0.3575	0.1140	0.1401	0.026*	
C7	0.25333 (16)	0.22777 (12)	0.25968 (16)	0.0207 (3)	
C8	0.05357 (14)	0.32340 (12)	0.16232 (17)	0.0193 (3)	
C9	−0.03152 (16)	0.31722 (13)	0.27635 (18)	0.0253 (4)	
H9	−0.0110	0.2681	0.3513	0.030*	
C10	−0.14675 (18)	0.38349 (15)	0.2797 (2)	0.0325 (4)	
H10	−0.2045	0.3798	0.3580	0.039*	
C11	−0.17880 (18)	0.45468 (14)	0.1711 (2)	0.0340 (4)	
H11	−0.2574	0.5002	0.1750	0.041*	
C12	−0.09518 (18)	0.45894 (13)	0.0565 (2)	0.0300 (4)	
H12	−0.1174	0.5067	−0.0193	0.036*	
C13	0.02102 (16)	0.39388 (13)	0.05141 (18)	0.0240 (3)	
H13	0.0782	0.3974	−0.0274	0.029*	
H1	0.204 (2)	0.2485 (15)	0.072 (3)	0.035 (6)*	

### Atomic displacement parameters (Å^2^)

**Table:**

	*U*^11^	*U*^22^	*U*^33^	*U*^12^	*U*^13^	*U*^23^
O1	0.0268 (6)	0.0522 (7)	0.0143 (6)	0.0088 (5)	−0.0021 (5)	−0.0028 (5)
N1	0.0190 (7)	0.0273 (6)	0.0138 (6)	0.0021 (5)	0.0007 (5)	−0.0007 (5)
C1	0.0241 (8)	0.0280 (8)	0.0241 (8)	0.0058 (6)	0.0028 (7)	0.0048 (7)
C2	0.0238 (9)	0.0310 (8)	0.0299 (9)	0.0038 (6)	0.0055 (7)	0.0028 (7)
C3	0.0240 (9)	0.0374 (9)	0.0418 (11)	0.0083 (7)	0.0003 (8)	0.0062 (8)
C4	0.0290 (9)	0.0291 (8)	0.0420 (10)	0.0068 (7)	−0.0009 (8)	0.0094 (8)
C5	0.0234 (8)	0.0268 (7)	0.0275 (8)	0.0017 (6)	−0.0010 (7)	0.0065 (7)
C6	0.0189 (7)	0.0242 (7)	0.0222 (7)	0.0028 (6)	−0.0003 (6)	0.0005 (6)
C7	0.0194 (7)	0.0238 (7)	0.0188 (7)	−0.0006 (6)	−0.0015 (6)	−0.0007 (6)
C8	0.0184 (7)	0.0198 (6)	0.0196 (7)	−0.0009 (5)	−0.0043 (6)	−0.0032 (6)
C9	0.0230 (8)	0.0303 (8)	0.0226 (8)	0.0025 (6)	−0.0014 (6)	−0.0008 (7)
C10	0.0226 (8)	0.0405 (9)	0.0345 (9)	0.0044 (7)	0.0008 (7)	−0.0063 (8)
C11	0.0249 (9)	0.0279 (8)	0.0493 (11)	0.0073 (6)	−0.0079 (8)	−0.0078 (8)
C12	0.0317 (10)	0.0219 (8)	0.0366 (9)	−0.0007 (6)	−0.0142 (8)	0.0017 (7)
C13	0.0232 (8)	0.0252 (7)	0.0236 (8)	−0.0040 (6)	−0.0063 (7)	0.0014 (7)

### Geometric parameters (Å, °)

**Table:**

O1—C7	1.226 (2)	C5—C6	1.530 (2)
N1—C7	1.353 (2)	C5—H5A	0.9900
N1—C8	1.4176 (19)	C5—H5B	0.9900
N1—H1	0.85 (3)	C6—C7	1.520 (2)
C1—C2	1.527 (2)	C6—H6	1.0000
C1—C6	1.529 (2)	C8—C9	1.390 (2)
C1—H1A	0.9900	C8—C13	1.395 (2)
C1—H1B	0.9900	C9—C10	1.389 (2)
C2—C3	1.526 (2)	C9—H9	0.9500
C2—H2A	0.9900	C10—C11	1.383 (3)
C2—H2B	0.9900	C10—H10	0.9500
C3—C4	1.527 (3)	C11—C12	1.385 (3)
C3—H3A	0.9900	C11—H11	0.9500
C3—H3B	0.9900	C12—C13	1.390 (2)
C4—C5	1.535 (2)	C12—H12	0.9500
C4—H4A	0.9900	C13—H13	0.9500
C4—H4B	0.9900		
			
C7—N1—C8	126.23 (15)	C6—C5—H5B	109.6
C7—N1—H1	116.4 (15)	C4—C5—H5B	109.6
C8—N1—H1	116.4 (14)	H5A—C5—H5B	108.1
C2—C1—C6	110.94 (12)	C7—C6—C1	111.75 (12)
C2—C1—H1A	109.5	C7—C6—C5	112.16 (13)
C6—C1—H1A	109.5	C1—C6—C5	110.46 (13)
C2—C1—H1B	109.5	C7—C6—H6	107.4
C6—C1—H1B	109.5	C1—C6—H6	107.4
H1A—C1—H1B	108.0	C5—C6—H6	107.4
C3—C2—C1	111.43 (14)	O1—C7—N1	122.67 (15)
C3—C2—H2A	109.3	O1—C7—C6	122.53 (14)
C1—C2—H2A	109.3	N1—C7—C6	114.80 (14)
C3—C2—H2B	109.3	C9—C8—C13	119.85 (14)
C1—C2—H2B	109.3	C9—C8—N1	122.21 (14)
H2A—C2—H2B	108.0	C13—C8—N1	117.94 (14)
C2—C3—C4	111.49 (14)	C10—C9—C8	119.40 (15)
C2—C3—H3A	109.3	C10—C9—H9	120.3
C4—C3—H3A	109.3	C8—C9—H9	120.3
C2—C3—H3B	109.3	C11—C10—C9	121.10 (18)
C4—C3—H3B	109.3	C11—C10—H10	119.4
H3A—C3—H3B	108.0	C9—C10—H10	119.4
C3—C4—C5	110.85 (13)	C10—C11—C12	119.33 (16)
C3—C4—H4A	109.5	C10—C11—H11	120.3
C5—C4—H4A	109.5	C12—C11—H11	120.3
C3—C4—H4B	109.5	C11—C12—C13	120.47 (17)
C5—C4—H4B	109.5	C11—C12—H12	119.8
H4A—C4—H4B	108.1	C13—C12—H12	119.8
C6—C5—C4	110.48 (14)	C12—C13—C8	119.83 (16)
C6—C5—H5A	109.6	C12—C13—H13	120.1
C4—C5—H5A	109.6	C8—C13—H13	120.1
			
C6—C1—C2—C3	−55.5 (2)	C1—C6—C7—N1	78.01 (17)
C1—C2—C3—C4	54.7 (2)	C5—C6—C7—N1	−157.32 (13)
C2—C3—C4—C5	−55.3 (2)	C7—N1—C8—C9	−32.8 (2)
C3—C4—C5—C6	56.9 (2)	C7—N1—C8—C13	148.20 (15)
C2—C1—C6—C7	−177.32 (14)	C13—C8—C9—C10	−1.4 (2)
C2—C1—C6—C5	57.07 (19)	N1—C8—C9—C10	179.54 (15)
C4—C5—C6—C7	176.87 (13)	C8—C9—C10—C11	0.6 (2)
C4—C5—C6—C1	−57.75 (17)	C9—C10—C11—C12	0.7 (3)
C8—N1—C7—O1	4.1 (2)	C10—C11—C12—C13	−1.1 (2)
C8—N1—C7—C6	−175.38 (13)	C11—C12—C13—C8	0.2 (2)
C1—C6—C7—O1	−101.43 (19)	C9—C8—C13—C12	1.0 (2)
C5—C6—C7—O1	23.2 (2)	N1—C8—C13—C12	−179.90 (14)

### Hydrogen-bond geometry (Å, °)

**Table:**

*D*—H···*A*	*D*—H	H···*A*	*D*···*A*	*D*—H···*A*
N1—H1···O1^i^	0.85 (3)	1.98 (3)	2.8145 (19)	171.7 (18)

Symmetry codes: (i) −*x*+1/2, *y*, *z*−1/2.
